# MINDFULNESS AND COGNITIVE FUNCTION IN PATIENTS WITH COGNITIVE IMPAIRMENT

**DOI:** 10.1093/geroni/igz038.2444

**Published:** 2019-11-08

**Authors:** Magdalena I Tolea, Juyoung Park, James Galvin

**Affiliations:** Florida Atlantic University, Boca Raton, Florida, United States

## Abstract

Mental health benefits of mindfulness, the attribute of being aware and present in the moment, have long been acknowledged. Mindfulness has also been linked to improved cognitive performance and improvements in AD neuropathology (↓hippocampal atrophy, ↑brain connectivity) in MCI or early-stage AD patients. This study was designed to: investigate the relationship between mindfulness and cognitive function in a patient population with varying degrees of cognitive impairment; identify the specific mindfulness components that provide benefits; and explore differences by sex and disease severity. Patients (N=112; 43% female; 77.0±7.7yrs; 11% cognitively normal, 27% MCI, and 67% dementia) attending a university-based dementia clinic were administered the Applied Mindfulness Process Scale (AMPS) and underwent neuropsychological testing. Cognition was linearly regressed on AMPS with adjustment for age, gender, education, and disease stage, in the entire sample and stratified by sex and stage. In fully adjusted models, higher mindfulness was associated with lower AD8 scores (β=-0.05±0.02(p = 0.003)), better animal naming (AN)(β=0.11±0.04(p = 0.008)), and faster TMA times (β=-0.72±0.32(p=0.025)). All three mindfulness factors (F1=decentering; F2=positive emotional regulation; F3=negative emotional regulation) were significantly linked to AD8, while F3 was not predictive of AN, and F1 was not predictive of TMA. In addition, mindfulness significantly predicted subjective cognitive impairment (SCI)(βF2AD8=-0.18±0.07(p=0.011)) and TMA in men βTMA=-1.14±0.42(p=0.011); βF2TMA=-2.63±1.26(p=0.043); βF3TMA=-2.74±1.12(p=0.019) and dementia patients (βF1AD8=-0.19±0.08(p=0.021); βF2AD8=-0.14±0.07(p=0.044); βTMA=-0.09±0.51(p=0.039)); and AN in women ((βAN=0.12±0.06(p=0.047);βF2AN=0.34±0.16(p=0.036)) and MCI patients (βAN=0.13±0.06, p=0.033; βF3AN=0.36±0.16(p=0.035)). Our findings suggest that effectiveness of mindfulness-based interventions may be enhanced by a focus on emotional regulation and sex- and stage-specific cognitive targets.

